# Statistical analysis of arthroplasty data: I. Introduction and background

**DOI:** 10.3109/17453674.2011.588862

**Published:** 2011-07-08

**Authors:** Jonas Ranstam, Johan Kärrholm, Pekka Pulkkinen, Keijo Mäkelä, Birgitte Espehaug, Alma Becic Pedersen, Frank Mehnert, Ove Furnes

**Affiliations:** ^1^Swedish National Competence Center Musculoskeletal Disorders, Skåne University Hospital, Lund, The Swedish Knee Arthroplasty Register, and Lund University; ^2^The Swedish Hip Arthroplasty Register, Sahlgrenska University Hospital and Göteborg University, Göteborg, Sweden; ^3^The Finnish Arthroplasty Register and Department of Public Health, University of Helsinki; ^4^The Finnish Arthroplasty Register and Turku University Central Hospital, Turku, Finland; ^5^The Norwegian Arthroplasty Register, Department of Orthopaedic Surgery, Haukeland University Hospital, Bergen, Norway; ^6^The Danish Hip and Knee Arthroplasty Register, Department of Clinical Epidemiology, Competence Center North, Aarhus University Hospital, Aarhus, Denmark; ^7^Department of Surgical Sciences, University of Bergen, Norway

## Abstract

It is envisaged that guidelines for statistical analysis and presentation of results will improve the quality and value of research. The Nordic Arthroplasty Register Association (NARA) has therefore developed guidelines for the statistical analysis of arthroplasty register data. The guidelines are divided into two parts, this one with an introduction and a discussion of the background to the guidelines, and the second one with a more technical statistical discussion on how specific problems can be handled (Ranstam et al. 2011b, see pages x-y in this issue). This first part contains an overview of implant survival analysis and statistical methods used to evaluate factors with a potential influence on this outcome.

In 1996, the guidelines known as the Consolidated Standards of Reporting Trials (CONSORT) Statement (Begg et al. 1996) were finalized. A few years earlier, two groups of experts on clinical trials had started to develop publication guidelines for randomized clinical trials, first independently of each other and later together. This was a reaction to the many reports from randomized clinical trials that had been published with insufficient information on items important for assessment of their quality.

Since then, reporting guidelines have also been developed for a number of other types of studies. Several journals (including Acta Orthopaedica) consider compliance with the guidelines to be compulsory and request that manuscripts be submitted together with completed guidelines checklists (Vandenbroucke 2009).

Methodological guidelines have been developed in parallel to reporting guidelines. The introduction of the ICH guideline “Statistical Principles for Clinical Trials”, adopted by the regulatory bodies of the European Union, Japan, and the USA in 1998, was, for example, the first time that clear and consistent regulatory guidance on statistical principles had been made available internationally.

The CONSORT reporting guidelines have clearly improved the reporting of clinical trials (Plint et al. 2006), and the “Statistical Principles for Clinical Trials” have had a “huge positive impact” on the quality of clinical trials by promoting a unified standard of good statistical practice (Brown et al. 2008).

Assuming that guidelines play an equally important role in improvement of the reliability and the value of registry research, the Nordic Arthroplasty Register Association (NARA) study group decided at a meeting in Lund, Sweden, in September, 2009, to develop statistical recommendations for analysis of arthroplasty data.

## Standard methods for analysis of survival data

The term “survival analysis” is used for statistical methods developed for data that define time intervals, with one starting point and one endpoint. In analyses of data from arthroplasty registers, the time intervals analyzed may represent the survival of implants, where the starting point is the date of the primary operation and the endpoint is the date of revision.

Usually, not all implants will be revised. Some implants are well-functioning, or at least unrevised at the end of the study, or they may have been implanted in a person who died or was lost to follow-up for some other reason. The survival times of such incomplete observations are called “censored”. Censored observations should also be included in the analysis, because even if the exact time of revision is not known, the implant is at least known to be unrevised before being censored.

It is usually assumed that censoring occurs at random. This may not always be the case, however. How departures from random censoring can be handled is discussed under the heading “Competing risk”, in Part II, Section 1.

Two functions are used to describe survival data: the survival function and the hazard function. The survival function (*S*(*t*)) is expressed as the probability that the survival time, for example of an implant, is greater than or equal to time *t*, and the hazard function (*h*(*t*)) can be expressed as the hazard (risk) of revision at some time *t* given that the implant has survived to that time without revision.

### Life table and Kaplan-Meier estimates

The estimated survival function is often presented as a table or a graph (survival curve). The survival function can be estimated using two different methods: the life table (actuarial) approach (Cutler and Ederer 1958) and the Kaplan-Meier (product-limit) approach (Kaplan and Meier 1958).

Both methods are non-parametric, i.e. no assumption regarding the distribution of survival times is required, and for both methods the survival function is calculated as the cumulative probability that an implant will survive through a set of time intervals.

The main difference between the methods is that while the life table is calculated for a set of predefined time intervals, the Kaplan-Meier method defines the time intervals to include only one event (revision) and is thus independent of a subjective choice of time intervals.

The probability of an implant surviving until time *t* (*S*(*t*)) is calculated as the probability of the implant surviving the first postoperative day multiplied by the probability of it surviving the second postoperative day given that the implant was not revised the first day, multiplied by the probability of it surviving the third postoperative day given that the implant was not revised the first or second day, and so on, until time *t*.

Assuming *k* revisions occurring at *k* different times during follow-up (*t*_1,_ *t*_2_, …., *t* *_k_* ≤ *t*), the survival until time *t* may thus be written as a product of conditional probabilities:


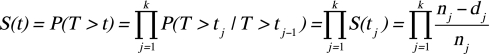


where *n* *_j_* = number at risk at revision time *t* *_j_* and *d* *_j_* = number of revisions at time *t* *_j_*.

If there are no censored observations, the Kaplan-Meier estimate of the survival function at time t will be equal to the proportion of implants unrevised at that time.

Confidence intervals may also be calculated for values of the survival function and these are often based on the Greenwood formula for calculation of standard errors. The life table method has been discussed (Dobbs 1980) and recommended in relation to arthroplasty data (Murray et al. 1993). However, the Kaplan-Meier method is usually preferred if the exact times of revision are known.

As an example, the Kaplan-Meier estimated survival functions for implants A, B, and C are given in the Figure. The survival probability at 20 years was 88% (95% CI: 87–89) for implant A, 81% (78–84) for implant B, and 92% (90–94) for implant C (Table 1).

**Figure:**
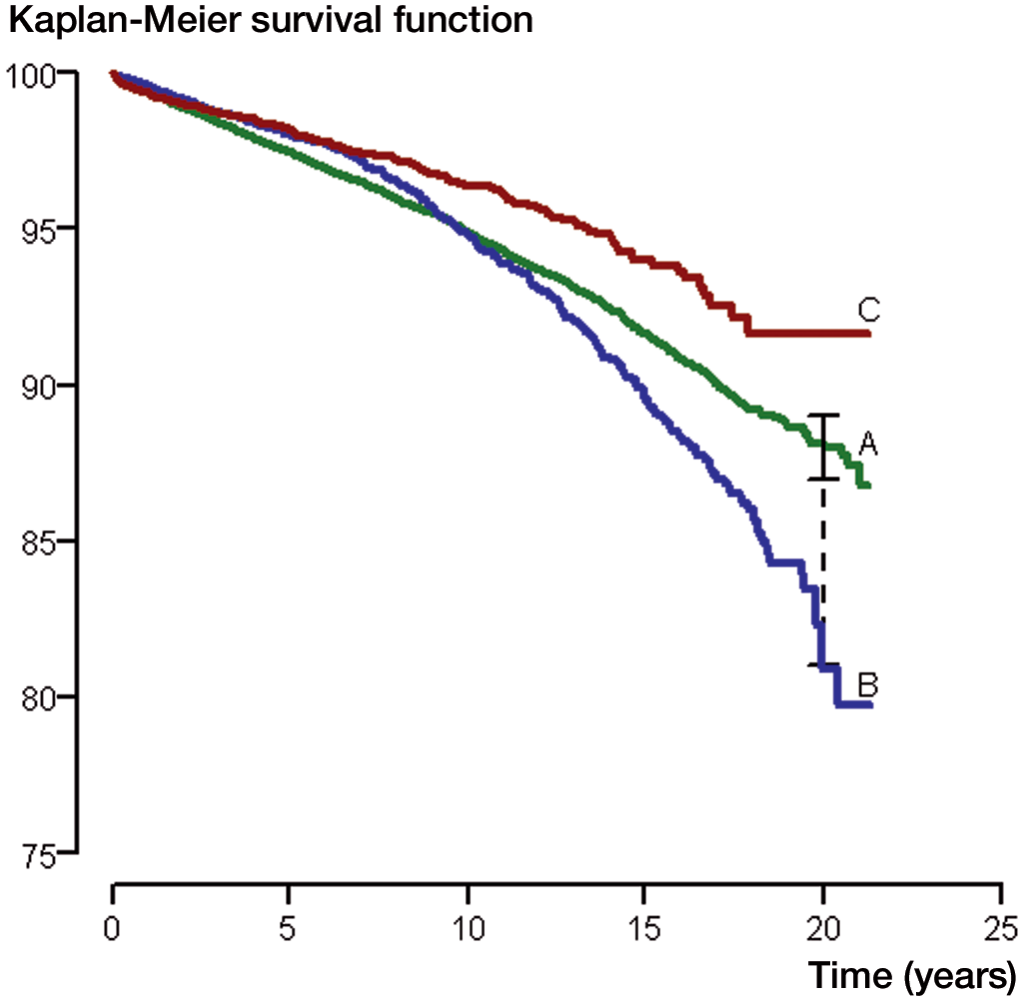
Kaplan-Meier survival curves for implants A, B, and C with standard (solid line) and modified (dotted line) 95% confidence limits for implant A at 20-year follow-up. In the modified CI the number of implants still at risk at the time of interest is taken into account in the calculation of the lower confidence limit.

**Table 1.** Kaplan-Meier estimated survival probabilities for implants A, B, and C with standard 95% confidence interval (CI) and modified confidence interval (CI_mod_) with the Peto lower confidence limit. With the CI_mod_, the number of implants still at risk at the time of interest is taken into account in the calculation of the lower confidence limit

15-year survival				20-year survival		
Implant	%	CI	CI_mod_	%	CI	CI_mod_
A	91	91–92	90–92	88	87–89	81–89
B	90	88–90	86–91	81	78–84	47–84
C	94	93–95	90–95	92	90–94	60–94

The confidence limits given are based on survival data not at 20 years, but at the time of the last revision before this time. It is clear that with heavy censoring, as might be the case towards the end of the survival curve, the number at risk may have decreased considerably after the time of the last revision and the estimated confidence intervals might therefore be too narrow.

Alternative methods have been suggested for obtaining confidence intervals based on the number of implants still at risk at the time of interest (Dorey and Korn 1987). The numbers given in Table 1 show that confidence limits modified in this way are much wider. Non-overlapping 95% confidence intervals imply a difference that is statistically significant at the 5% level. A small degree of overlap does not preclude the possibility of statistical significance, however.

### Log-rank test

In this example, the difference in survival is relatively small (2.3%, 95% CI: 1.2–3.4), and it is based on only one time point (15 years)—which may not be representative of the total follow-up. It is also a problem that the investigators' subjective choice of width of time interval can affect the estimated difference in survival between the implants.

The log-rank test (Mantel and Haenszel 1959), on the other hand, is based on the total follow-up, and it tests the null hypothesis of there being no difference in the survivor functions of 2 (or more) implants.

In our example, this test gave a p-value of less than 0.001, which indicates that the difference in estimated survival was not due to chance.

The log-rank test is known by several other names, and there are many variations of the test. In contrast to the log-rank test, which puts equal weight on all uncensored observations, the Gehan test weights each uncensored observation by the number of implants still at risk (*n*) at the time of failure (Gehan 1965). This test will therefore put more weight on what happens early after implantation. Tarone and Ware (1977) suggested that weights equal to the square root of n would give a more efficient test.

### Cox regression

In observational studies, such as registry studies, there may be systematic differences between groups of patients with different types of implants, and these systematic differences may affect the validity of the results by confounding bias. For example, if more high-risk patients receive implant A than implant B, a crude comparison of the 2 implants may show that implant A, despite being just as good as implant B, appears to have shorter survival.

Also, in well-designed randomized controlled trials there may be imbalances between study groups due to chance. Several strategies, or combinations of strategies, exist that can be used to adapt to this situation (Havelin et al. 2004). These include selection of a more homogenous material and adjustments by the Cox proportional hazards regression model (Cox 1972).

The Cox model is semi-parametric and assumes no particular distribution for the survival times. It is, however, based on the assumption that the hazard of implant A at any given time is proportional to the hazard of implant B. This is known as the proportional hazards (PH) assumption.

Survival curves that cross, as for implant A and B in the Figure, imply that the PH assumption is violated. The consequences of such departures from the assumption, methods to diagnose them, and how such departures can be handled are discussed in detail under the heading “The proportional hazards assumption” in Part II, Section 2.

The previously discussed log-rank test is also based on an assumption of PH.

In the Cox model, the hazard function is defined as *h(t)* = h_0_(t)e^β*X^, where the baseline hazard function *h_0_(t)* (common to all implants included) is multiplied by a factor representing the revision risk associated with some factor *X* (e.g. implant type).

The exponential function ensures positive values for the hazard function. The corresponding survival function is written as


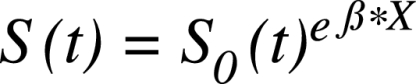


(*S_0_(t)* being the baseline survival function).

If *X* is defined as an indicator variable, equal to 0 when implant A has been used and 1 when implant B is used, the hazard ratio—often interpreted as the relative risk (RR) for implant B versus implant A with respect to revision—can be written:


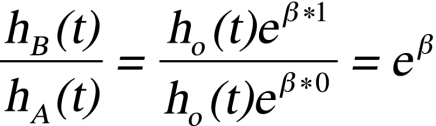


In the example, the estimated hazard ratio (or RR) is 1.08 (95% CI: 0.98–1.19) when implant B is compared to implant A, and 0.72 (0.62–0.83) when comparing implant C with A (p < 0.001). This suggests that implant B has a hazard rate that is 8% higher than that of implant A, while the hazard rate of implant C is 72% of that of implant A (Table 2). The 95% confidence intervals describe the uncertainty in these estimates.

**Table 2.** Cox regression estimated crude and adjusted relative risk (RR) of revision comparing implant B and C with implant A. Breakdown of patient gender and age according to type of implant

			Simple Cox regression			Multiple Cox regression **^a^**		
Implant	% males	% ≥ 70 years	RR	95 % CI	p-value	RR	95 % CI	p-value
A	29	61	1			1		
B	28	64	1.08	0.98–1.19	0.1	1.05	0.95–1.16	0.3
C	27	78	0.72	0.62–0.83	< 0.001	0.80	0.69–0.93	0.003

**^a^** Adjustment for age (≤ 59, 60–69, 70–79, ≥ 80), sex, and diagnosis (OA, other).

In the same way, one could also say that the estimated hazard of implant A is 1.4 (i.e. 1/0.72) times higher than that of implant C.

Risk estimates such as these, estimated without any inclusion of covariates in the statistical model, are often called crude or unadjusted. Thus, the risk estimates discussed above do not include adjustment for systematic imbalance in predictive factors between the implant groups.

To account for such factors and to increase the validity of the result, the Cox model can be expanded to include also covariates representing known or suspected confounders:


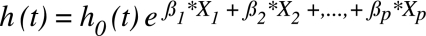


For example, if information on patient age, sex, and primary diagnosis is included in the model, the hazard ratio for the implant type will be estimated conditionally on these factors, i.e. the risk estimate will be protected from the effects of imbalance in these factors, adjusted for confounding by association with the factors included in the model. Successful adjustment, however, requires that all confounding factors be identified and measured—and often relies on assumptions of log-linear effects. It is conceivable that confounding effects may remain after adjustment. This is known as residual confounding.

In the example, the new adjusted estimate comparing implant C with implant A was 0.78 (95% CI: 0.69–0.92; p = 0.002), meaning that the relative difference was somewhat reduced by the adjustment (Table 2).

The difference in crude and adjusted estimates was due to the fact that a larger proportion of older patients with better prognosis had received implant C than implant A. Table 2 also shows that the crude and adjusted relative risk estimates for comparison of implant B against implant A were similar.

The Cox regression model thus is a tool to explore the effect of one or more factors on survival and to adjust for confounding factors. Identification of confounders may be a problem when covariates are highly correlated.

Furnes et al. (2001) observed that several diagnoses found in younger patients gave reduced implant survival compared to primary osteoarthritis of the hip, but that the effect of hip disease disappeared with adjustment for age and use of uncemented implants with inferior survival. Since uncemented implants were mostly used for younger patients, it was difficult to ascertain which of these factors was the most important confounder. Here, it was helpful to study the effect of hip disease in subgroups defined by age and prosthesis use. Another important reason for doing subgroup analysis is to investigate whether the impact of a risk factor on implant survival differs among subgroups (interaction). The precision of these results will, however, depend on the number of patients in each stratum. An alternative could be to include the interaction terms in a new multiple model.

It should also be noted that the selection of covariates to be included in the statistical model for confounding adjustment requires careful considerations regarding cause and effect. The decision should not be based on p-values from p-value screenings of crude effects or on the results of an automatic stepwise regression method.

While Kaplan-Meier survival curves illustrate crude or unadjusted differences in implant survival, results from Cox regression analyses can be used to construct prosthesis-specific survival curves with adjustment for relevant factors such as age and sex. These are often based on Cox regression analyses with implant brand as stratification factor and the curves calculated for mean values of the other factors included in the model. The way to do this is debated, however, and other methods have been suggested (Ghali et al. 2001, Cole and Hernán 2004).

## Other methods of survival analysis

Other methods for analysis of survival exist. The best known in the context of arthroplasty register data is probably Poisson regression, which is often used to combine results from different studies in meta-analyses, but other methods are also available: parametric survival models, accelerated failure time models, and Aalen's linear regression. These methods will, however, not be discussed here.

## Other methodological problems

Arthroplasty register data have several characteristics that may have special consequences for the precision and validity of the results from statistical analyses. One such issue is bilaterality.

### Bilateral observations

One basic assumption in the statistical methods that are most often used to analyze arthroplasty register data is that observations are independent. This assumption is not fulfilled when bilateral observations are included in the analysis. Two observations from the same patient can be assumed to be correlated, i.e. that within-subject variance differs from between-subject variance.

While this may theoretically have consequences for the precision and validity of the results, no one has shown this to be a practical problem for analyses of arthroplasty register data (Robertsson and Ranstam 2003, Lie et al. 2004). How the statistical analysis should deal with this issue is addressed in Part II, Section 3.

### Revision rate ranking

Arthroplasty registers often have a prominent role in evaluations of quality of healthcare and in comparisons of counties and hospitals. The results from statistical analyses using the previously described methods—for example, revision rates and adjusted relative revision risks—are then used as benchmarks, as criteria for ranking, and for compilation of league tables.

However, the observed revision rate may seem clear and objectively measured but it is, from a statistical standpoint, uncertain because of unavoidable sampling errors and measurement (or registration) errors.

While manifestations of sampling uncertainty can be identified by the variability in results from multiple samples used for the same estimation (for example, the replicates of a laboratory experiment), it may be more difficult to recognize sampling uncertainty in a single sample—which is often the case for hospital comparisons.

Revision rates from different hospitals are therefore often compared directly (Ranstam et al. 2008), without any consideration for the fact that the estimates being compared are uncertain. The consequences of this, and suggestions for assessment and presentation of the uncertainties for ranking of revision risk estimates are discussed in Part II, Section 4.
